# Testing the predictive value of functional traits in diverse ant communities

**DOI:** 10.1002/ece3.10000

**Published:** 2023-04-19

**Authors:** Kim I. Drager, Michael D. Rivera, Joshua C. Gibson, Selina A. Ruzi, Priscila E. Hanisch, Rafael Achury, Andrew V. Suarez

**Affiliations:** ^1^ Department of Evolution, Ecology and Behavior University of Illinois 505 S. Goodwin Ave. 515 Morrill Hall Urbana Illinois 61801 USA; ^2^ Program in Ecology, Evolution and Conservation Biology University of Illinois 505 S. Goodwin Ave. 515 Morrill Hall Urbana Illinois 61801 USA; ^3^ Department of Entomology University of Illinois 505 S. Goodwin Ave. 320 Morrill Hall Urbana Illinois 61801 USA; ^4^ Beckman Institute for Advanced Science and Technology University of Illinois at Urbana‐Champaign 405 N. Mathews Ave Urbana Illinois 61801 USA; ^5^ Department of Applied Ecology North Carolina State University 115 David Clark Labs, 100 Eugene Brooks Avenue Raleigh North Carolina 27695 USA; ^6^ Department of Animal Ecology and Tropical Biology Biocenter University of Würzburg Am Hubland 97074 Würzburg Germany; ^7^ Museo Argentino de Ciencias Naturales “Bernardino Rivadavia” MACN‐CONICET Buenos Aires Argentina; ^8^ Terrestrial Ecology Research Group Technical University of Munich Hans‐Carl‐von‐Carlowitz‐Platz 2 Freising 85354 Germany

**Keywords:** *d*N, Formicidae, morphology, phylogeny, stable isotopes, trophic position

## Abstract

Associating morphological features with ecological traits is essential for understanding the connection between organisms and their roles in the environment. If applied successfully, functional trait approaches link form and function in an organism. However, functional trait data not associated with natural history information provide an incomplete picture of an organism's role in the ecosystem. Using data on the relative trophic position of 592 ant (Formicidae) samples comprising 393 species from 11 subfamilies and 19 widely distributed communities, we tested the extent to which commonly used functional proxies (i.e., morphometric traits) predict diet/trophic position as estimated from stable isotopes (δ15N). We chose ants as a group due to their ubiquity and abundance, as well as the wealth of available data on species traits and trophic levels. We measured 12 traits that have previously been identified as functionally significant, and corrected trait values for size and evolutionary history by using phylogenetically corrected trait residuals. Estimated trophic positions varied from 0.9 to 4.8 or roughly 4 trophic levels. Morphological data spanned nearly the entire size range seen in ants from the smallest (e.g., *Strumigenys mitis* total length 1.1 mm) to the largest species (e.g., *Dinoponera australis* total length 28.3 mm). We found overall body size, relative eye position, and scape length to be informative for predicting diet/trophic position in these communities, albeit with relatively weak predictive values. Specifically, trophic position was negatively correlated with body size and positively correlated with sensory traits (higher eye position and scape length). Our results suggest that functional trait‐based approaches can be informative but should be used with caution unless clear links between form and function have been established.

## Graphical abstract

Identifying and associating morphological features with ecological traits are essential for understanding the connection between organisms and their roles in the environment. Using data on the relative trophic position of 592 ant (Formicidae) samples comprising 393 species from 11 subfamilies and 19 widely distributed communities, we tested the extent to which commonly used functional proxies (i.e., morphometric traits) predict diet/trophic position as estimated from stable isotopes (δ15N). We found overall body size, relative eye position, and scape length to be informative for predicting diet/trophic position in these communities, albeit with relatively weak predictive values.

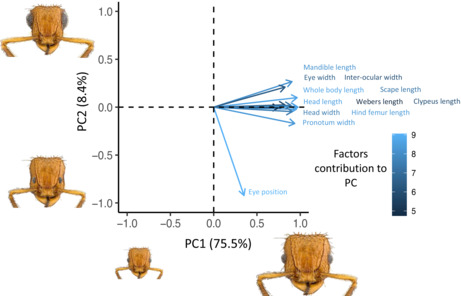

## 1 INTRODUCTION

Variation in morphology reflects the distinct ways organisms interact with each other and the environment. Morphological traits therefore provide valuable, mechanistic insights into the diverse ecological strategies that organisms use to survive and co‐exist under differing environmental conditions (Westoby, 1998; Westoby & Wright, 2006). Trait‐based approaches have a long history in ecology (McGill et al., 2006; Weiss & Ray, 2019), and their application has increased considerably in recent years (Wong et al., 2019). However, for many organisms, there is often a disconnect between functional traits (i.e., features linked to an ecological strategy that influence fitness) and functional groups (i.e., common ecological strategies among organisms; Sobral, 2021; Violle et al., 2007). In some groups like plants, functional traits are categorized based on clear ecological roles (e.g., nitrogen fixers; Cornelissen et al., 2003; Ledeganck et al., 2003), but in other taxa, these links are not well established. Yet, there is an increasing use of morphological trait data as a stand‐in for ecological function in analyses of “functional diversity” (Pigot et al., 2020; Sobral, 2021).

Using morphological traits to categorize communities assumes that species with similar traits perform similar roles in their community or are exposed to common environmental filters. However, correlations between traits and functional outcomes should remain hypothetical unless paired with causal evidence or detailed natural history information. Further, functional outcomes are the result of complex ecological interactions not easily compared across communities (i.e., many ways to reach the same trophic position), and both biotic and abiotic factors can generate convergence and divergence of traits (Cadotte & Tucker, 2017). Finally, misleading correlations between traits and ecological function may be found if shared evolutionary histories are not accounted for (Flynn et al., 2011). Tests of trait‐based approaches in community ecology should take these limitations into account and ideally use data from taxonomically diverse species across multiple communities when possible (Weiss & Ray, 2019).

In recent years, there have been many studies speculating/proposing functional traits in animals, particularly terrestrial arthropods (reviewed in Wong et al., 2019). A variety of traits have been identified that are hypothesized to play a role in ecological function through processes such as feeding niche or response to abiotic stress (Moretti et al., 2017). Using a framework developed for plants based on responses to stress and disturbance, Andersen (1995) classified Australian ants within communities into discrete functional groups. These groups defined the ecological roles of species through characteristics such as high activity and domination of resources (Dominant Dolichoderine) and the narrow range of environments or microhabitats they inhabit (Climate Specialists; Andersen, 1995). While primarily based on taxonomic divisions and competitive hierarchies, this effort set the stage for functional approaches to the study of ant ecology globally (Andersen, 1995; Gibb et al., 2015; Parr et al., 2017). For example, this classification was applied to North American communities (albeit with taxonomically different species; e.g., Moranz et al., 2013) and has been widely used in neotropical communities (Silvestre et al., 2003). While useful in comparing broad community structures, care should be taken to not confound functional groups with having shared functional traits. More recent approaches often correlate trait morphospace with ecology to examine functional diversity (e.g., Nooten et al., 2019; Retana et al., 2015; Scharnhorst et al., 2021). However, conclusions from linking environmental or biological variation to morphological features of the species living there remain tenuous due to untested assumptions about traits and their function (but see Gibb & Parr, 2013; Nooten et al., 2019; Retana et al., 2015).

Several studies implicate traits in determining ecological function in ants (Davidson et al., 2004; Sarty et al., 2006; Table 1). For example, aspects of eye morphology have been used both as indicators of predatory behavior (positively correlated) and hypogeal activity (negatively correlated) (Jelley & Barden, 2021; Narendra et al., 2013; Weiser & Kaspari, 2006). While morphological traits such as Weber's length (i.e., longest axis of the mesosoma) and pronotum width are not directly linked to fitness, such traits nonetheless are considered “proxy traits,” serving as indicators of ant performance (Arnold, 1983; Sarty et al., 2006; Violle et al., 2007; Weber, 1938). Although traits like these have been used in various ant studies, the links between morphological traits and ecological function are rarely well understood, particularly at broader ecological and phylogenetic scales (Gibb et al., 2015). A robust test of the universality of linking traits to a specific function like diet would require comparing the trophic position of species to their morphological traits or morphospace values across diverse communities and environments.

**TABLE 1:** Traits measured for each ant species including description of measurement, its suggested functional significance, and an image of how it was measured.

Trait (abbreviation)	Description	Source(s)	Example
Worker body size proxies correlate with metabolic level, used habitat complexity, mandible musculature			
Weber's length (WL)	With body in lateral view, the length for a straight line between the point at which the pronotum meets the cervical shield and the posterior basal angle of the metapleuron	Weber (1938)	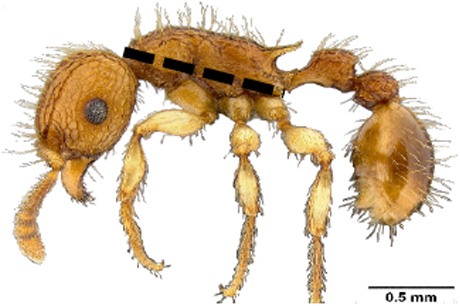
Head length (HL)	With head in dorsal view, the length of a straight line drawn across the head of the ant at its longest point, including lobes but excluding spines and mandibles	Sarty et al. (2006)	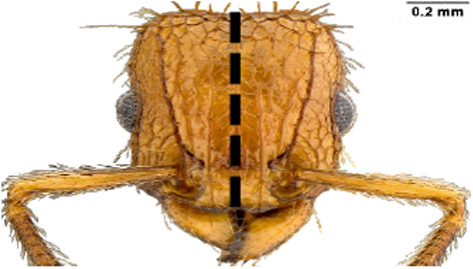
Head width (HW)	With head in dorsal view, the length of a straight line drawn across the head of the ant at its widest point, including eyes		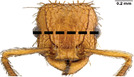
Pronotum width (PW)	With body in dorsal view, the length of the pronotum at its widest point, excluding spines	Sarty et al. (2006)	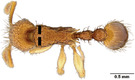
Body length (TBL)	With body in lateral view, the sum of the length of the left mandible, head capsule, WL, petiole and postpetiole (when present), and gaster		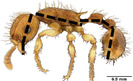
Traits that impact foraging performance, foraging speed, habitat use, prey size, and liquid feeding rate			
Hind femur length (HFL)	The square root of the sum of the squared length of the hind femur with the body in dorsal view and the squared height of the hind femur with the body in lateral view	Feener Jr et al. (1988); Sommer & Wehner (2012)	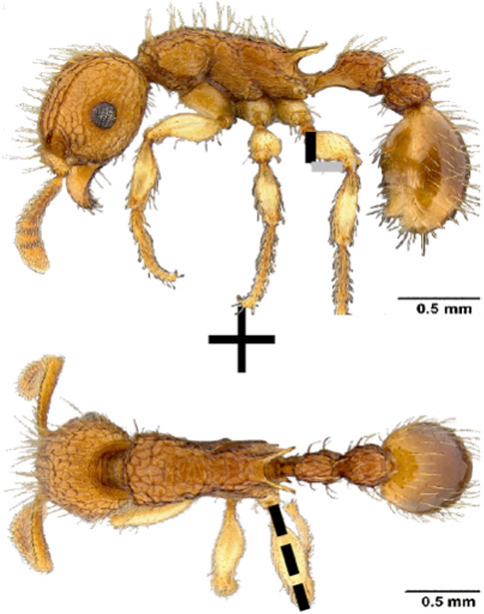
Mandible length (ML)	With head in dorsal view, the length of the straight line drawn between the distal most tooth of the left or right mandible, depending on which is most visible, and the midpoint of its base	Fowler et al. (1991); Gibb & Cunningham (2013)	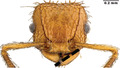
Clypeus length (CL)	With head in dorsal view, the length of a straight line between the dorsal and anterior margins of the clypeus at its widest point	Davidson et al. (2004)	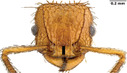
Sensory traits affecting foraging strategy, foraging location, habitat utilization			
Scape length (SL)	The square root of the sum of the squared length of the left or right scape in dorsal view and the squared height of the scape with either the head in full face view or the body in lateral view, depending on the position of the antennae and of the ant on the point	Feener Jr et al. (1988); Sommer and Wehner (2012)	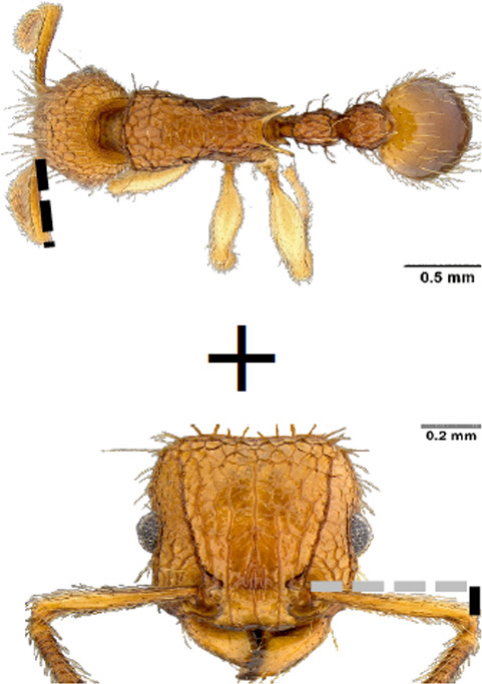
Inter‐ocular width (IOW)	With head in dorsal view, the minimum distance between the medial margins of the compound eyes, when present	Weiser & Kaspari (2006)	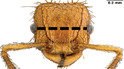
Max eye width (EW)	With body in lateral view, the length of the widest point of the left compound eye	Fowler et al. (1991); Gibb & Parr (2013)	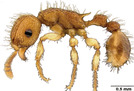
Eye position (EP)	Ratio of the distance shortest distance from the anterior most margin of the left compound eye in lateral view and HL	Weiser & Kaspari (2006)	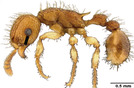

In this study, we ask whether the position in functional morphospace predicts trophic position in a taxonomically diverse dataset of ants within and among 19 sites across a broad biogeographic range. To do this, we used published data on the relative trophic position of ants within and among diverse ecological communities. Ants are an ideal study system due to their ubiquity and abundance, as well as the wealth of available data on species traits and estimated trophic position based on the use of stable isotopes of Nitrogen (𝛿15N). Nitrogen isotopic values are typically enriched by 3 to 4 ‰ between trophic levels, a pattern established across a variety of arthropod taxa including ants (Blüthgen et al., 2003; Minagawa & Wada, 1984; Tillberg et al., 2006). We control for both shared evolutionary history and scaling effects using a genus‐level phylogeny and ordination of 12 size‐corrected morphological traits. We tested the following two hypotheses based on predictions from the literature (Gibb et al., 2015; Jelley & Barden, 2021; Weiser & Kaspari, 2006; Yates et al., 2014). First, if large worker size benefits prey capture, trophic position will be positively correlated with body size. Alternately, if body size is limited by energy availability, trophic position will be negatively correlated with body size due to the increased use of plant‐based resources by larger species (Wills et al., 2018). Second, traits associated with sensory systems (e.g., eye size and position) or prey capture (e.g., mandible length) will have higher predictive power for species at higher trophic positions (Jelley & Barden, 2021; Weiser & Kaspari, 2006). Finally, a high degree of specialization within some clades (e.g., army ants of the subfamily Dorylinae) can result in strong phylogenetic niche conservatism (Losos, 2008). We therefore examined the phylogenetic signal of morphometric traits and trophic position across the entire Family and within individual subfamilies.

## 2 METHODS

### 2.1 Data selection

We searched the literature for studies where stable isotopes were used to infer the relative trophic position of species within their ecologically diverse communities. We targeted studies that examined communities of ants rather than those that focused on single/few taxa to avoid possible taxonomic biases and to provide as broad a comparison as possible among species in the context of the communities they reside. We selected seven papers that sampled ant communities from natural environments, estimated their relative trophic position using stable isotopes of N including the use of plant samples to base this inference, and identified samples to species or morphotypes if specimens were available to measure. We rejected studies that experimentally manipulated nutritional resources or focused on a single species/clade in a community (i.e., leaf‐cutting ants). If values were not provided in the paper, isotopic data were extracted from figures using the *digitize* package (version 0.0.4, Poisot, 2011) in R (version 3.4.2, R Core Team, 2017). Trophic position was calculated using an equation from Post (2002):

(1) $$\text{Trophic level}=\lambda +\frac{\left(D15N\;\text{Secondary consumer}-D15N\;\text{base}\right)}{\delta N},$$

where λ = primary producer, and δN = trophic step = 3.4 permil.

For each paper included in our analyses, we compiled a list of ants examined in each study. Ants not identified as species were removed from our dataset except for Tillberg et al. (2007) where voucher specimens could be obtained to measure morphological traits (see below).

### 2.2 Measurements

Representative images of the full body in dorsal view, full body in lateral view, and head in dorsal view of one specimen for each species in this list were downloaded from AntWeb (2020). Only one imaged specimen per species was included because there are a limited number of imaged specimens per species available on AntWeb. In instances where multiple specimens were imaged, we chose which specimen to measure based on the following criteria, in decreasing order of significance: (1) if the species had a polymorphic worker caste and more than one caste was imaged, the minor caste was given priority over the major caste; (2) if two specimens differed in the number of traits that we could measure based on the positioning of the ant in the frame, the specimen with the greater number of measurable traits was given priority; (3) specimens collected from localities geographically closer to the site from the study in question were given priority; and (4) specimens collected closer to the date that the study in question was conducted were given priority. We measured 12 traits that have previously been considered ecologically important (Table 1; Yates et al., 2014; Sosiak & Barden, 2021). A flowchart showing how data were captured, curated, and analyzed is in Figure S1 and the data compiled from the literature or generated here is in Dataset S1.

All measurements taken from images were calculated using ImageJ (Version 1.52a, Ferreira & Rasband, 2011). For all species in Hanisch et al. (2020) and a subset of morphotypes specimens included in the Tillberg et al. (2007) dataset, the measurements were taken on physical specimens rather than images using a Semprex Micro‐DRO digital stage micrometer (Semprex Corp.) attached to a Leica MZ 12.5 stereomicroscope and a LEICA M165C stereomicroscope with a LEICA DFC295 camera. A complete list of CASENT numbers corresponding to the specimens measured from AntWeb is in Dataset S1.

### 2.3 Cleaning the data and creating phylogeny

Samples with duplicate names were given a unique identification and for the purpose of this study were treated as a separate species. We used the genus‐level phylogeny from Blanchard and Moreau (2017). Several samples did not have a generic representation in the phylogeny (*Gigantiops*, *Cladomyrma*, *Echinopla*, and *Proatta*). These taxa were added to the phylogeny using the *bind.tip* function in the *phytools* package (Revell, 2012; version 0.7.85) in R. Their placement was determined using Ward (2014) and Blaimer et al. (2018), and their divergent time was set at half the current branch's length. From this phylogeny, we added species into genus‐level polytomies using the *genus.to.species.tree* and *collapse.to.start* functions in the *phytools* package (Revell, 2012).

### 2.4 Missing data and size correction

Due to either the nature of AntWeb images (e.g., not all features are fully in frame) or damaged voucher specimens, we were unable to measure every morphological trait for all species leading to some missing data (Figure S2). Therefore, to compute our principal component analysis (PCA), we first impute missing morphological data. We used a principal component analysis model implemented in the *missMDA* R package (version 1.18; Josse & Husson, 2016) using the *estim_ncpPCA* and *imputePCA* functions. These estimated traits allowed us to perform further analyses with a complete dataset with minimal impact on future PCAs.

We corrected trait values for overall size by using phylogenetically corrected trait residuals using the *phytools* function *phyl.resdi*. This performs a phylogenetic generalized least‐square regression (PGLSR) on each trait with Weber's length and calculates the residuals for each sample and trait. While this method uses a phylogenetically informed regression, these residual values are not “phylogenetically corrected” as values are not altered to reflect the effects of divergence time using an evolutionary model (Revell, 2009). Given many of these traits are known to be correlated with body size, we present primarily the results using trait residuals. Additionally, we also analyze the raw trait values, labeled when presented.

### 2.5 Estimating phylogenetic signal

We looked for a phylogenetic signal in morphological traits and estimated trophic position using multiple metrics, including Pagel's Lambda, Bloomberg's K, Moran's I, and Abouheif's C_mean_ index, implemented with the *phylosignal* R package (Abouheif, 1999; Blomberg et al., 2003; Keck et al., 2016; Pagel, 1992), and tested the null hypothesis that the morphological traits and trophic values are randomly distributed across the phylogeny. Phylogenetic signal was estimated using the size‐corrected trait residual, imputed datasets, and raw morphological measurements. We also measured K_mult_, a multivariate generalization of Bloomberg's K for the combined morphological dataset (Adams, 2014).

### 2.6 PCA space and correlations with trophic position

We compared both individual traits and a combined morphospace value (PCA loading) to trophic position. While correlations to individual traits are relatively straightforward to interpret, the morphospace approach provides a more holistic approach that has been applied to ants (Weiser & Kaspari, 2006). To explore and describe the PCA we used the *phyl.pca* function in the *phytools* package under a Brownian motion model. To further describe the morphospace, we grouped samples into categorical trophic positions. A phylogenetic PCA was performed to characterize morphological space only, further analysis of principal components (PCs) vs trophic position utilized a nonphylogenetic PCA as phylogeny is corrected for using PGLSR, thus evolutionary relationships were accounted once for each analysis. Additional components were added if the amount of variation explained increased by at least 5%. We estimated the correlation between trophic position and trait values using a PGLSR for the whole dataset, by subfamily, and by country (see Table S4). In each case, the phylogeny was pruned to the available dataset. We present adjusted alpha values for multiple comparisons to control for false discovery rates (Benjamini & Yekutieli, 2001, implemented in *p.adjust* in the *stats* package), when comparing individual traits with trophic position and when comparing PCA to trophic value by subfamily. To measure the relationship between PC values and trophic position we used a PGLSR.

### 2.7 Phylogenetic flexible discriminate analysis

In addition to comparing PC values with trophic position, we also used discriminate analyses to determine the utility of morphological traits in classifying specimens into trophic levels (defined as a whole integer of the isotopic values, 1–5). We used phylogenetic flexible discriminate analysis, a combination of phylogenetic generalized linear regression with a flexible discriminate analysis (Motani & Schmitz, 2011). The resulting classifications can be compared with observed groupings via a confusion matrix, summarizing misclassifications. These were performed on size‐corrected data with missing data imputed. We used the *phylo.fda* function from Motani & Schmitz, 2011) to perform the PFDA and create the confusion matrix.

## 3 RESULTS

### 3.1 Data set summary

**FIGURE 1:**
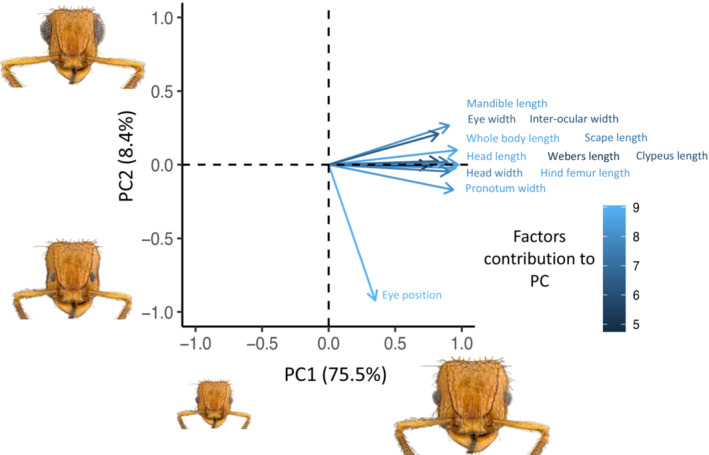
Size‐corrected principal component analysis (PCA) loadings—these loadings are products of the PCA that are used in regression with trophic position. Variable color represents contribution of each variable to the PC. Even size‐corrected, we see many traits that are associated with size group together; thus, PC1 generally describes size. PC2 is primarily influenced by eye position. The ant heads illustrate these general patterns: size increases with PC1; eye and scape size get larger; and eyes are positioned wider apart and higher on the head with PC2.

We compiled data from 7 papers, that had trophic information for 592 specimens across 19 communities (Table 2). We combined isotopic data with morphological measurements for 446 specimen records and 347 unique species across these studies. For most species, all morphological traits were measured (<10% missing data due to damaged specimens or poor specimen positioning in images for most measurements; Figure S2). Relative isotopic values varied from 0.92 to 4.82, or roughly 4 trophic levels, which is consistent with the dietary range of ants (Tillberg et al., 2007, 2014). The phylogenetic PCA with size‐corrected traits had loadings on two principal components (PCs; Table 3, Figure 1). PC1 included most traits related to body size, with traits missing more data contributing less (i.e., clypeus length), and explained ~75% of the variance in the morphospace. PC2 was primarily driven by eye position and explained ~8.5% of the morphospace. Further principal components explained less than 5% of the additional variation (Figure S3).

**TABLE 2:** Articles including community‐wide isotopic values of ants whose data were used in the analyses.

Reference		Biogeographic region
Blüthgen et al. (2003)	https://doi.org/10.1007/s00442‐003‐1347‐8	Australia. (North Queensland)
Davidson et al. (2003)	https://doi.org/10.1126/science.1082074	South America and Asia. (Peru and Brunei)
Fiedler et al. (2007)	https://doi.org/10.1007/s00040‐007‐0959‐0	Central Europe (Austria, Germany, and Switzerland)
Gibb et al. (2015)	https://doi.org/10.1007/s00442‐014‐3101‐9	Australia (South‐eastern Australia)
Pfeiffer et al. (2014)		Malaysia (Sarawak)
Hanisch et al. (2020)	https://doi.org/10.1111/een.12817	South America (Misiones, Argentina)
Tillberg et al. (2007)	https://doi.org/10.1073/pnas.0706903105	South and North America (Argentina and United States)

**TABLE 3:** Results from the phylogenetic size‐corrected, generalized least‐square regression on individual traits vs. trophic value at the family level with (A) size‐corrected data and (B) raw trait values.

	*n*	Intercept	Slope	SE	*p*‐value	Sigma
(A) Trait (trait size residual)						
Head width	442	5.499	−1.185	0.531	.0273	13.03
Head length	441	5.483	−0.5118	0.7023	.4666	13.11
Mandible length	429	5.473	0.8014	0.9731	.4106	13.23
Clypeus length	425	5.543	−1.413	1.1335	.2133	13.24
**Interocular width**	**410**	**5.207**	**−1.721**	**0.5399**	**.0015**	**13.35**
**Body length**	**421**	**5.501**	**−0.7619**	**0.1527**	**9.00E‐07**	**12.97**
Eye width	406	5.229	−5.457	2.2748	.0168	13.35
Eye position	396	5.223	−0.6157	0.9089	.4985	13.39
Pronotum width	431	5.485	0.2091	0.7735	.7870	13.21
Hind femur length	393	5.42	0.6759	0.6054	.2649	13.61
Scape length	388	5.483	1.626	0.6722	.0160	13.62
(B) Trait (raw trait value)						
Head width	**443**	**4.639**	**0.5513**	**0.0993**	**4.89E‐08**	**18.51**
Head length	**442**	**4.735**	**0.4206**	**0.0952**	**1.25E‐05**	**18.76**
Mandible length	**430**	**5.602**	**−0.3675**	**0.0908**	**6.17E‐05**	**19.04**
Clypeus length	**426**	**5.715**	**−0.7278**	**0.1882**	**1.28E‐04**	**19.18**
Inter‐ocular width	411	5.425	−0.3634	0.1584	2.23E‐02	19.72
Weber's length	**444**	**4.579**	**0.3855**	**0.0504**	**1.34E‐13**	**17.98**
Body length	**422**	**5.715**	**−0.05987**	**0.0179**	**9.41E‐04**	**19.32**
Eye width	**407**	**5.42**	**−1.726**	**0.4161**	**4.11E‐05**	**19.38**
Eye position	**396**	**4.197**	**1.769**	**0.5572**	**1.62E‐03**	**19.9**
Pronotum width	**432**	**5.869**	**−0.5432**	**0.1494**	**3.11E‐04**	**19.09**
Hind femur length	**393**	**5.93**	**−0.3358**	**0.0648**	**3.55E‐07**	**19.47**
Scape length	**390**	**5.075**	**0.2971**	**0.0854**	**5.65E‐04**	**19.45**

*Note*: The phylogeny was pruned to match the available dataset, and bold values reflect significance where alpha adjusted to *p* = .0045 and .00416 for A and B, respectively.

Abbreviation: SE, standard error.

### 3.2 Phylogenetic signal

Across morphological traits, we see significant effects of phylogeny on trait distributions, with the strongest association with mandible length (multiple metrics, see Table S2). The multivariate phylogenetic signal was estimated to be 0.3791 and was significant based on 1000 random permutations (*p* = .001, effect size = 15.1288, Figure S4). Trophic value was estimated to have a moderate phylogenetic signal (Abouheif's Cmean = 0.5275, Moran's I = 0.1008, Pagel'sλ = 0.74, Blomberg's *K* = 0.39; all estimates were significant based on 1000 random permutations).

### 3.3 Individual traits vs trophic position

Size‐corrected head width/length, inter‐ocular width, eye width, scape length, and whole‐body length all had significant negative relationships with trophic position, with eye width having the steepest negative slope across the whole family Formicidae (PGLS regression, *p* < .001). Only inter‐ocular width and whole‐body length maintain their significance with a false discovery rate adjusted *p*‐value (Table 3A). The impact of scaling is evident when comparing raw trait values to trophic position as nearly all the traits show significant relationships with trophic position (Table 3B). When broken down by subfamily, several other traits had significant negative (mandible length in Dolichoderines) and positive (Weber's length in dolicoderinaes and myrmicines) correlations with trophic position (PGLS regression, *p*‐values <.05) but only whole‐body length in myrmicines remained significant after alpha adjustment (Table 4). Examining ant communities from each country separately, traits varied with trophic position more sporadically, with only seven significant site‐specific correlations primarily related to body size (Weber's length—Malaysia; whole‐body length—Malaysia, Argentina, Australia; pronotum width—Australia; clypeus length—Argentina; eye position—Argentina; Table S4).

**TABLE 4:** Summary of significant correlations (+ positive, − negative) between traits (size‐corrected trait residuals) and trophic position (note: sample sizes varied across clade).

	HW	HL	ML	CL	IOW	WL	TBL	EW	EP	PW	HFL	SL
Formicidae	−				−*		−*	−				+
Formicinae					−							
Dolichoderinae			−	−								
Myrmicinae			−		−		−*		−			
Ponerinae									+			
Dorylinae									+			

*Note*: See Table S1 for specific values.

Trait abbreviations are described in Table 1.

* Indicates significance via an adjusted alpha of 0.0045; others are significant with an alpha of 0.05.

### 3.4 Morphological PC vs trophic position

**FIGURE 2:**
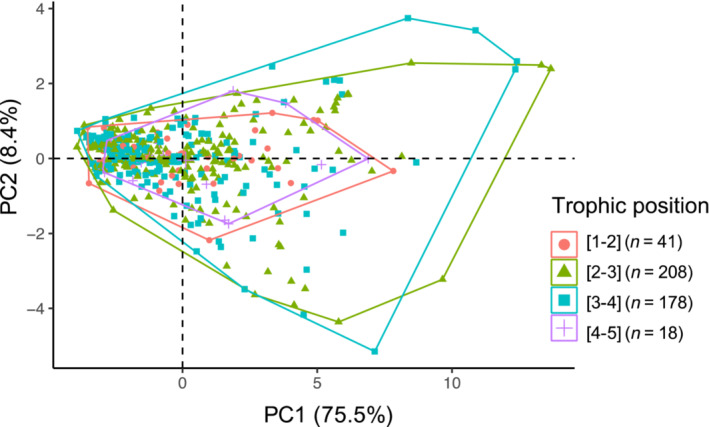
Size‐corrected principal component analysis (PCA) loadings colored by 4 categorical groupings of relative trophic position (1–2 = herbivore, 2–4 = omnivore/primary predator, 4–5 = top predator). These points from a standard PCA are used in the regression with tropic position.

**FIGURE 3:**
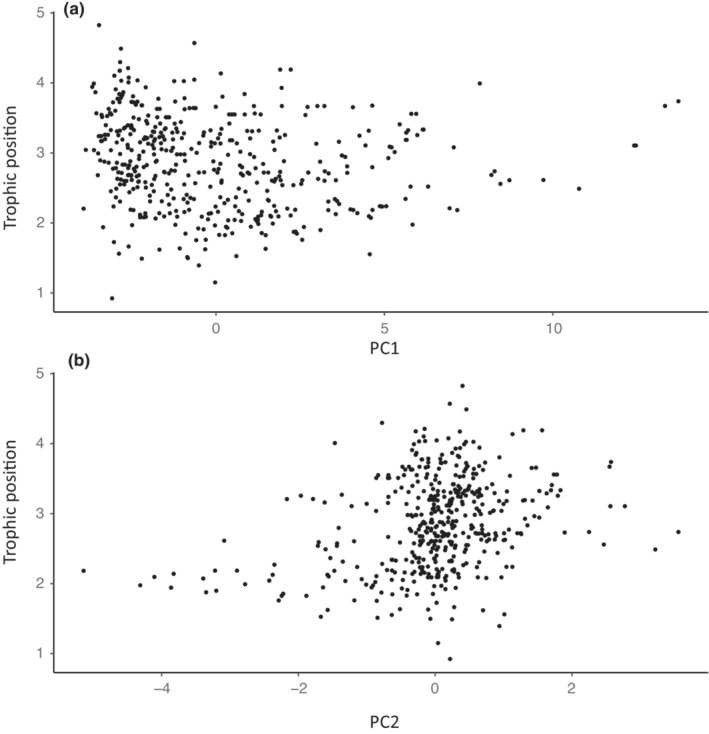
Phylogenetic generalized least‐square regression of trophic position against size‐corrected PC1 (a) and PC2 (b). Both principal components and trophic position are phylogenetically corrected under a Brownian motion model of trait evolution. Across all samples, there was no significant correlation between size‐corrected morphological PC1 and PC2 values with trophic position (PGLS: *t* = −1.949, *p* = .052).

Viewing trophic position categorically, highly predatory species occupy a smaller (but not unique) portion of the morphospace (Figure 2). Herbivorous and omnivorous samples spread out from the origin, following the first two principal components. Across all samples, there was no significant correlation between size‐corrected morphological PC1 and PC2 values with trophic position (PGLS: *t* = −1.949, *p* = .052; Figure 3a,b). Analyzed by subfamily, only two showed significant correlations between PC1 and trophic position: Myrmicinae (PGLS: *p* = .003) and Pseudomyrmcinae (PGLS: *p* = .026). There was no correlation between PC2 and trophic position at the family level (PGLS *t* = −1.32, *p*‐value = .188). The subfamily Formicinae had a positive correlation between PC2 and trophic position while Dolichoderine and Ponerinae had negative correlations (Table 4, Table S1).

### 3.5 Phylogenetic flexible discriminate analysis

The pFDA showed high levels of trophic level misclassification using morphological traits (λ=1.0, misclassification error = 0.544, *n* = 588). The model had difficulty distinguishing among the omnivores of moderate trophic level, frequently classifying species of trophic level four as level three (Table S3a). These results were robust to the level of phylogenetic signal used, tested using lambda values ranging from zero to one, and misclassifying more than half of the species in all iterations.

## 4 DISCUSSION

A goal of functional traits is to connect variation in measurable morphological features with variation in an organism's role in an ecosystem. Once validated they can then be useful proxies for measuring the response of communities across spatial and temporal scales (Drenovsky et al., 2013; Hoenle et al., 2023). We sought to test the link between frequently measured morphological traits and a key ecological role, trophic position. Overall, we found support that certain morphological traits related to body size and eye position are informative to predict trophic position. However, many morphological traits that are traditionally used in functional trait‐based approaches for ants did not show evidence of functional prediction with respect to the relative trophic position in several widely distributed ant communities.

We found several traits at the family level correlated with trophic position, particularly traits related to overall body size (HW, TBL), and to sensory organs including eye size (EW, IOW) and scape length. The antenna is the primary way that ants interact with their environment. The positive relationship between antenna length (scape length) and trophic position could reflect the need for predatory ants to detect prey at a distance and to facilitate prey capture. Features of the eye play an important role in foraging in ants (Jelley & Barden, 2021), perhaps most emblematic is the active predator *Gigantiops destructor* whose large eyes facilitate jumping while hunting (Beugnon et al., 2001). Functionally larger eyes could allow foragers to track and locate moving prey, an advantage over the chemically based foraging of herbivores and detritivores ants, thus representing a higher trophic position (Fowler & Delabie, 1995). However, applying this functional link across Formicidae can confound the effects on eye size of predation with the relaxed constraints in species that live or forage primarily below ground (i.e., hypogeic). Hypogeous species are noted for their small eyes, similar to the ocular reduction in troglodytic taxa (Rétaux & Casane, 2013). Yet, these are often highly predacious such as some army ants in the subfamily Dorylinae (Hoenle et al., 2019) or the genus *Hypoponera* in the subfamily Ponerinae (Hanisch et al., 2020).

Our results suggest that bigger species tend towards lower relative trophic positions than smaller ones, a pattern seen in many animals. This could be explained by the metabolic and ecological restrictions associated with body size and its effects on access to food resources (Farji‐Brener et al., 2004). However, a positive relationship between body size and prey size is also often predicted within food webs, and this pattern is seen in the subfamily Ponerinae, which consists of many large, predatory species (Hanisch et al., 2020). Applying a functional trait framework to body size in ants requires extra considerations for several reasons. First, by being social, ants may overcome morphological constraints with behavioral adaptations. For example, small ants may subdue larger moving prey by working together. Body size may therefore interact with colony size, an important trait that varies widely in ants and that can be hard to measure (Burchill & Moreau, 2016). Second, ants interact with the world differently based on their size (Kaspari & Weiser, 1999). Smaller ants may move through the leaf litter as if negotiating mountains, while larger ants with long legs will simply walk over it. Therefore, the functional significance of traits may be size and microhabitat‐dependent (Hoenle et al., 2023). Third, approximately 16% of ant species exhibit worker polymorphism, where a differential larval environment fosters the development of workers of different sizes and/or shapes (Wills et al., 2018). This size variation can have functional consequences (e.g., foraging speed, prey selection, behavioral dominance; Retana et al., 2015) thus worker variation may influence trophic position. Our size‐corrected PCA suggests that a number of trait residuals are still correlated with body size, meaning that larger individuals have relatively larger traits. Positive allometry is common in ant morphology, and these traits may be more likely to have functional significance. Moreover, some polymorphic species may have clearly defined functional roles (i.e., vertebrate defense in *Eciton* army ants or high‐efficiency foragers in leaf cutter ants).

Trait space occupied by species that vary in trophic position did not reveal distinct clusters but a nested relationship with taxa at the high (top predators) and low (near consumers) trophic levels occupying less morphospace than generalists. This could suggest that there are many ways of being a generalist, but dietary specialists are more morphologically constrained. However, this pattern appears driven by relatively few morphological outliers and may reflect differences in overall species number in each category rather than true constraints. Similar results from the pFDA show considerable overlap in middle trophic positions, leading to misclassifications in our model. Functional studies of these outlying species linking their morphology, diet, and natural history would be valuable.

It may prove difficult to establish global or family‐wide functional traits for ants, particularly for trophic position. While there has been no comprehensive survey of ant diets, our results suggest a sea of generalist species, with islands of specialized species, shown in the paucity of trophic extremes. Many species are scavengers foraging on any number of living/dead animal tissue and nitrogen‐poor plant‐based resources (both directly and those harvested from mutualist partners). These foraging habitats, along with possible measurement error, may explain some of the low estimates for trophic position we obtained where ants overlapped with primary producers in their communities. Plasticity in diet may also be influenced by colony needs and demography, local variation in nutrient availability, and variation in other local biotic and abiotic factors (Kaspari et al., 2012; Roeder & Kaspari, 2017). When analyzed at multiple geographic scales, we find few significant correlations between traits and trophic position (Tables S4 and S5) suggesting that in addition to trophic position, these functional relationships may also be geographically heterogeneous. We can therefore expect trophic position to vary across time and space even within most species. An exception may be highly specialized species whose diets are accompanied by morphological and social adaptations (e.g., leaf‐cutting ants, army ants). Studies of these specialized species may prove valuable in establishing functional links as their traits may show strong selection on performance. Even so, some taxa that appear to be specialized predators, like trap‐jaw ants with their power‐amplified mandibles, are often revealed to incorporate plant‐based material into their diet (e.g., Evans & Leston, 1971), weakening the relationship between morphology and function. Studies on endosymbiotic bacteria in ants have also revealed significant microbial contributions to nutrition in some groups (Russell et al., 2009), which could further complicate correlates of morphology with trophic position.

### 4.1 Limitations

Morphological traits are clearly constrained by forces other than feeding ecology within ants and among other arthropods (Retana et al., 2015; Wong et al., 2019). We see significant levels of phylogenetic signal, suggesting closely related species share similar morphological measurements than by chance. Additionally, behavioral adaptations may work to overcome morphological constraints. For example, small ants that may be overpowered by larger moving prey could overcome their small size by working together to subdue and ultimately move prey back to their nest. Future work on this topic should explicitly examine how morphology interacts with colony size and foraging behavior. By measuring only one specimen per species, we were also unable to capture intraspecific variation, and our measurements could be biased if the worker measured is somehow not representative of species means or the species was highly polymorphic. While measuring more ants would increase the probability of obtaining accurate species‐level measurements (Gaudard et al., 2019 recommend measuring at least 6 individuals) we were constrained by the number of usable photographs uploaded to AntWeb. Given the number of species involved, the number of sites considered, and the wealth of stable isotope data used to estimate relative trophic positions, this data set provides a robust test of how reasonable it is to assume associations between ecology and morphology. Future efforts that include a more comprehensive examination of the rich stable isotope data available for ants would allow for other links to be established, for example, between morphology, diet, and environmental variation. Additional data will also help address any biases that may have arisen from the specific papers we chose and how trophic position estimates were standardized across studies.

## 5 CONCLUSION

There are numerous applications of properly linked functional traits. From a conservation standpoint, identifying and preserving functional diversity may be a useful tool for identifying target species or areas for preservation, and for predicting responses to environmental change (Guilherme et al., 2019; Pigot et al., 2020). Additionally, understanding how traits shape communities will help us better predict how environmental change will affect community composition (Wellstein et al., 2011). Here we considered a large number of species, sites, and stable isotope data to estimate relative trophic positions to provide a robust test of how reasonable it is to assume associations between ecology and morphology in ants. We found support for body size and two sensory traits (scape length and eye position/size) having a predictive value for relative trophic position in ants. These results stress the functional value of traits that are involved in how ants interact with their environment. However, without additional studies linking form and function, a functional trait framework may not be generally applicable for ants, suggesting that the interpretation of functional trait analyses should be evaluated cautiously. Moreover, the patterns we found may also not be broadly applicable to other arthropod groups especially those whose natural history information is even less available than in ants.

## AUTHOR CONTRIBUTIONS

**Kim I. Drager:** Conceptualization (equal); data curation (equal); formal analysis (equal); investigation (equal); writing – original draft (equal); writing – review and editing (equal). **Michael D. Rivera:** Conceptualization (equal); data curation (equal); formal analysis (equal); investigation (equal); methodology (equal); writing – original draft (equal); writing – review and editing (equal). **Joshua C. Gibson:** Conceptualization (equal); data curation (equal); formal analysis (equal); methodology (equal); writing – original draft (equal); writing – review and editing (equal). **Selina Ariel Ruzi:** Conceptualization (equal); data curation (equal); formal analysis (equal); investigation (equal); methodology (equal); writing – original draft (equal); writing – review and editing (equal). **Priscila E. Hanisch:** Conceptualization (equal); data curation (equal); formal analysis (equal); investigation (equal); methodology (equal); writing – original draft (equal); writing – review and editing (equal). **Rafael Achury:** Conceptualization (equal); data curation (equal); methodology (equal); project administration (equal); writing – original draft (equal); writing – review and editing (equal). **Andrew Suarez:** Conceptualization (equal); formal analysis (equal); investigation (equal); methodology (equal); project administration (equal); supervision (equal); writing – original draft (equal); writing – review and editing (equal).

## FUNDING INFORMATION

National Science Foundation, (Grant Number: 'NSF DEB 17‐01501', 'NSF IOS 17‐55336').

## CONFLICT OF INTEREST STATEMENT

The authors have no conflict of interest.

## Supporting information


Dataset S1

Appendix S1

## Data Availability

All data are available as supplementary material in this publication.
