# Correction to “Testing the predictive value of functional traits in diverse ant communities”

**DOI:** 10.1002/ece3.11134

**Published:** 2024-03-18

Drager, K.I., M.D. Rivera, J.C. Gibson, S.A. Ruzi, P.E. Hanisch, R. Achury, and A.V. Suarez. 2023. Testing the predictive value of functional traits in diverse ant communities. *Ecology and Evolution* 13: e10000. https://doi.org/10.1002/ece3.10000

The summary of the statistics for subfamily relationships between traits and trophic position in Table 4 is incorrect. The Table should have included the following significant correlations based on the results in Table S1:

**TABLE 4:** Summary of significant correlations (+ positive, − negative) between traits (size‐corrected trait residuals) and trophic position (note: sample sizes varied across clade).

	HW	HL	ML	CL	IOW	Web	WBL	EW	EP	Pro	HFL	SL
Formicidae												
Formicinae									−			
Dolichoderinae			−						+			
Myrmicinae			−*			−		−*		+*		
Ponerinae			+		−			−*	+*	+		+
Dorylinae						−	−					

*Note*: See Table S1 for specific values. *Indicates significance via an adjusted alpha of .0045, others are significant with an alpha of .05. Trait abbreviations are described in Table 1.

In addition, the description of the subfamily results in Section *3.3 Individual traits* vs. *trophic position* results section was incorrect. This should have read: “When broken down by subfamily, several other size‐corrected traits had significant negative (mandible length in Dolichoderines, Myrmicinae; eye position in Formicinae) and positive (Pronotum width in Myrmicinae and Ponerinae, eye position in Ponerinae) correlations with trophic position (PGLS regression, *p*‐values < .05) but only mandible length, eye width, and pronotum width in Myrmicinae, and eye width and position in Ponerinae remained significant after alpha adjustment (Table 4).”

We apologize for this error.

