# Supplementary material for: Testing the predictive value of functional traits in diverse ant communities
Source: Ecol Evol. 2023 Apr 19;13(4):e10000. doi: 10.1002/ece3.10000 (PMC10115899; doi:10.1002/ece3.10000)
Supplement: Supplementary file 2 — Appendix S1 [file ECE3-13-e10000-s002.docx]

Supplementary Figure 1. Flowchart of approach including description of methodology used to clean and analyze the dataset.


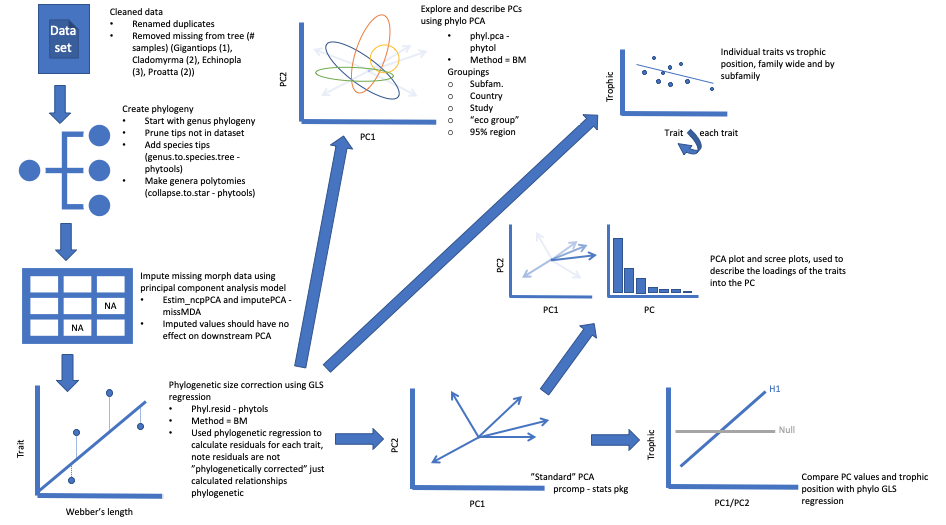


Supplementary Figure 2. Summary of missing morphological trait data of 589 observations across 12 morphological traits. All observations are associated with isotopic and trophic position data.

Supplementary Figure 3. Skree plot from the Standard PCA - shows the loading of variance for each of the successive principal components.


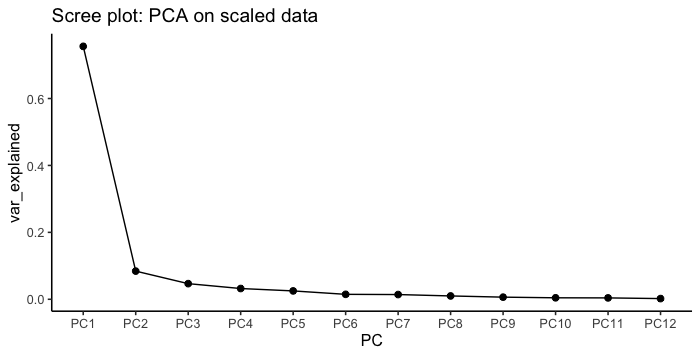


Principal component

Proportion of variation explained

Supplementary Figure 4. Histogram of phylogenetic signal estimate, Kmult from 1000 random permutation of the data, observed K value marked with an arrow.

Supplementary Table 1. Results from the phylogenetic size-corrected, generalize least squares regression on individual traits vs trophic value by subfamily. Note these are size corrected. The phylogeny was pruned to match the available dataset. SE = standard error

| **Trait** | **Subfamily** | **n** | **Intercept** | **Slope** | **SE** | **p-value** | **Adjusted p-value** |
| --- | --- | --- | --- | --- | --- | --- | --- |
| Head width | Formicinae | 93 | 5.00 | 0.05 | 0.80 | 0.95 | 0.98 |
| Head width | Dolichoderinae | 49 | 5.67 | -2.43 | 2.36 | 0.31 | 0.53 |
| Head width | Myrmicinae | 168 | 6.63 | 1.00 | 0.53 | 0.06 | 0.21 |
| Head width | Ponerinae | 68 | 5.71 | -1.05 | 01.00 | 0.30 | 0.52 |
| Head width | Dorylinae | 29 | 5.58 | -5.21 | 3.35 | 0.13 | 0.33 |
| Head length | Formicinae | 93 | 4.88 | -0.16 | 1.16 | 0.89 | 0.98 |
| Head length | Dolichoderinae | 49 | 5.67 | -1.28 | 2.35 | 0.59 | 0.84 |
| Head length | Myrmicinae | 169 | 6.64 | -0.86 | 0.62 | 0.17 | 0.38 |
| Head length | Ponerinae | 67 | 5.71 | -1.62 | 0.98 | 0.10 | 0.30 |
| Head length | Dorylinae | 29 | 5.58 | -4.19 | 4.57 | 0.37 | 0.58 |
| Mandible length | Formicinae | 92 | 4.88 | -0.06 | 2.31 | 0.98 | 0.98 |
| Mandible length | Dolichoderinae | 48 | 5.67 | -7.84 | 3.24 | 0.02 | 0.11 |
| Mandible length | Myrmicinae | 161 | 6.61 | -4.42 | 1.37 | 0.001 | 0.02 |
| Mandible length | Ponerinae | 66 | 5.67 | 2.07 | 0.91 | 0.03 | 0.13 |
| Mandible length | Dorylinae | 28 | 5.56 | -1.23 | 4.56 | 0.79 | 0.97 |
| Clypeus length | Formicinae | 92 | 4.88 | -0.09 | 1.60 | 0.96 | 0.98 |
| Clypeus length | Dolichoderinae | 48 | 5.67 | -3.84 | 2.36 | 0.11 | 0.30 |
| Clypeus length | Myrmicinae | 159 | 6.60 | -0.98 | 1.59 | 0.54 | 0.79 |
| Clypeus length | Ponerinae | 65 | 5.67 | 3.41 | 1.82 | 0.07 | 0.22 |
| Clypeus length | Dorylinae | 27 | 6.37 | 6.29 | 4.58 | 0.18 | 0.38 |
| Inter ocular width | Formicinae | 92 | 4.88 | -0.48 | 1.28 | 0.71 | 0.91 |
| Inter ocular width | Dolichoderinae | 47 | 5.64 | -0.77 | 3.25 | 0.81 | 0.97 |
| Inter ocular width | Myrmicinae | 155 | 6.61 | 1.13 | 0.59 | 0.06 | 0.21 |
| Inter ocular width | Ponerinae | 64 | 5.71 | -1.58 | 0.61 | 0.01 | 0.11 |
| Inter ocular width | Dorylinae | 21 | 5.17 | -0.42 | 4.14 | 0.92 | 0.98 |
| Weber’s length | Formicinae | 94 | 10.75 | 1.62E+14 | 3.93E+14 | 0.68 | 0.89 |
| Weber’s length | Dolichoderinae | 49 | 5.43 | -3.1E+14 | 6.48E+14 | 0.63 | 0.85 |
| Weber’s length | Myrmicinae | 169 | 2.17 | -1.3E+15 | 5.46E+14 | 0.02 | 0.11 |
| Weber’s length | Ponerinae | 68 | -0.53 | 4.92E+14 | 1.05E+15 | 0.64 | 0.85 |
| Weber’s length | Dorylinae | 29 | 4.36 | -1.6E+15 | 6.47E+14 | 0.02 | 0.11 |
| Whole body length | Formicinae | 88 | 4.97 | -0.38 | 0.42 | 0.37 | 0.58 |
| Whole body length | Dolichoderinae | 47 | 5.64 | 0.72 | 1.49 | 0.63 | 0.85 |
| Whole body length | Myrmicinae | 162 | 6.61 | -0.36 | 0.32 | 0.26 | 0.49 |
| Whole body length | Ponerinae | 64 | 5.68 | -0.05 | 0.18 | 0.78 | 0.97 |
| Whole body length | Dorylinae | 28 | 5.56 | -1.90 | 0.84 | 0.03 | 0.13 |
| Eye width | Formicinae | 88 | 5.29 | -0.52 | 3.21 | 0.87 | 0.98 |
| Eye width | Dolichoderinae | 48 | 5.67 | 13.23 | 11.37 | 0.25 | 0.48 |
| Eye width | Myrmicinae | 154 | 6.50 | -15.43 | 4.61 | 0.001 | 0.02 |
| Eye width | Ponerinae | 65 | 5.74 | -6.91 | 2.51 | 0.008 | 0.09 |
| Eye width | Dorylinae | 20 | 5.02 | -26.61 | 16.56 | 0.13 | 0.33 |
| Eye position | Formicinae | 86 | 5.12 | -2.56 | 1.07 | 0.02 | 0.11 |
| Eye position | Dolichoderinae | 46 | 5.58 | 8.53 | 4.11 | 0.04 | 0.17 |
| Eye position | Myrmicinae | 155 | 6.59 | 0.11 | 1.50 | 0.94 | 0.98 |
| Eye position | Ponerinae | 61 | 5.66 | 4.99 | 0.92 | < 0.001 | < 0.001 |
| Eye position | Dorylinae | 19 | 4.94 | 8.28 | 5.56 | 0.15 | 0.36 |
| Pronotum width | Formicinae | 91 | 4.99 | -0.07 | 1.18 | 0.95 | 0.98 |
| Pronotum width | Dolichoderinae | 48 | 5.67 | 3.37 | 5.23 | 0.52 | 0.78 |
| Pronotum width | Myrmicinae | 164 | 6.61 | 2.23 | 0.45 | < 0.001 | < 0.001 |
| Pronotum width | Ponerinae | 65 | 5.67 | 4.00 | 1.70 | 0.02 | 0.11 |
| Pronotum width | Dorylinae | 29 | 5.58 | -15.30 | 10.08 | 0.14 | 0.34 |
| Hind femur length | Formicinae | 84 | 4.88 | -0.94 | 1.00 | 0.35 | 0.58 |
| Hind femur length | Dolichoderinae | 39 | 5.62 | -2.25 | 1.64 | 0.18 | 0.38 |
| Hind femur length | Myrmicinae | 148 | 6.61 | 1.80 | 1.03 | 0.08 | 0.26 |
| Hind femur length | Ponerinae | 65 | 5.69 | 0.99 | 0.60 | 0.10 | 0.30 |
| Hind femur length | Dorylinae | 28 | 5.66 | 0.09 | 2.10 | 0.96 | 0.98 |
| Scape length | Formicinae | 71 | 4.84 | 1.13 | 1.28 | 0.38 | 0.58 |
| Scape length | Dolichoderinae | 45 | 5.56 | 0.44 | 1.97 | 0.82 | 0.97 |
| Scape length | Myrmicinae | 147 | 6.60 | -0.84 | 0.79 | 0.29 | 0.52 |
| Scape length | Ponerinae | 66 | 5.67 | 1.44 | 0.61 | 0.02 | 0.11 |
| Scape length | Dorylinae | 29 | 5.58 | -4.77 | 3.99 | 0.24 | 0.48 |

Supplementary Table 2. Phylogenetic signal on size-corrected, data imputed morphological measurements. All estimates were significant (p < 0.05) when estimated values are compared to repeated randomizations.

|  | Abouheif’s C | Moran’s I | Bloomberg’s K | Pagel’s Lambda |
| --- | --- | --- | --- | --- |
| Head width | 0.4407 | 0.0615 | 0.2946 | 0.7944 |
| Head length | 0.4502 | 0.0708 | 0.3423 | 0.8645 |
| Mandible length | 0.5249 | 0.0634 | 0.5308 | 0.9233 |
| Clypeus length | 0.3310 | 0.0493 | 0.3946 | 0.8795 |
| Inter ocular width | 0.3990 | 0.0556 | 0.2520 | 0.7187 |
| Weber’s length | 0.4835 | 0.0820 | 0.3643 | 0.8453 |
| Whole body length | 0.4808 | 0.0713 | 0.4050 | 0.8903 |
| Eye width | 0.4984 | 0.0756 | 0.3491 | 0.8446 |
| Eye position | 0.3522 | 0.0690 | 0.2415 | 0.6149 |
| Pronotum width | 0.4426 | 0.0697 | 0.2686 | 0.7542 |
| Hind femur length | 0.4068 | 0.0588 | 0.2879 | 0.8185 |
| Scape length | 0.4364 | 0.0688 | 0.3284 | 0.8826 |

Supplementary Table 3. Confusion matrix from the phylogenetic flexible discriminate analysis (pFDA). Bolded values are those classified correctly.

|  |  | **True trophic level** | | | | |
| --- | --- | --- | --- | --- | --- | --- |
| **Predicted trophic level** |  | 1 | 2 | 3 | 4 | 5 |
|  | 1 | **0** | 0 | 0 | 0 | 0 |
|  | 2 | 0 | **0** | **0** | 0 | 0 |
|  | 3 | 1 | 56 | **249** | 225 | 22 |
|  | 4 | 0 | 2 | 12 | **19** | 2 |
|  | 5 | 0 | 0 | 0 | 0 | **0** |

Supplementary Table 4. Results from the phylogenetic size-corrected, generalize least squares regression on individual traits vs trophic value by country. The phylogeny was pruned to match the available dataset. SE = standard error. Bolded rows represent relationships that remained significant at the country level.

| **Trait** | **Country** | **n** | **Intercept** | **Slope** | **Std error** | **P value** |
| --- | --- | --- | --- | --- | --- | --- |
| Head width | Malaysia | 80 | 3.39 | 0.230 | 0.160 | 0.154 |
| Head width | Argentina | 156 | 3.10 | 0.177 | 0.119 | 0.137 |
| Head width | Peru | 69 | 2.73 | -0.216 | 0.182 | 0.240 |
| Head width | Australia | 66 | 3.08 | -0.155 | 0.110 | 0.164 |
| Head width | Brunei | 49 | 2.60 | 0.092 | 0.096 | 0.341 |
| Head width | USA | 21 | 2.45 | 0.087 | 0.730 | 0.905 |
| Head length | Malaysia | 80 | 3.39 | -0.090 | 0.251 | 0.719 |
| Head length | Argentina | 157 | 3.05 | 0.174 | 0.148 | 0.240 |
| Head length | Peru | 70 | 2.73 | -0.311 | 0.181 | 0.091 |
| Head length | Australia | 66 | 3.08 | -0.194 | 0.133 | 0.150 |
| Head length | Brunei | 46 | 2.57 | 0.040 | 0.132 | 0.763 |
| Head length | USA | 21 | 2.45 | -0.650 | 0.939 | 0.497 |
| Mandible length | Malaysia | 79 | 3.39 | 0.023 | 0.316 | 0.941 |
| Mandible length | Argentina | 157 | 3.05 | 0.306 | 0.219 | 0.163 |
| Mandible length | Peru | 62 | 2.73 | 0.034 | 0.265 | 0.896 |
| Mandible length | Australia | 66 | 3.08 | -0.053 | 0.123 | 0.667 |
| Mandible length | Brunei | 43 | 2.63 | -0.133 | 0.306 | 0.665 |
| Mandible length | USA | 21 | 2.45 | 0.515 | 1.332 | 0.703 |
| Clypeus length | Malaysia | 77 | 3.39 | 0.397 | 0.311 | 0.205 |
| **Clypeus length** | **Argentina** | **152** | **3.02** | **0.801** | **0.314** | **0.011** |
| Clypeus length | Peru | 65 | 2.73 | 0.070 | 0.617 | 0.910 |
| Clypeus length | Australia | 66 | 3.08 | -0.159 | 0.159 | 0.320 |
| Clypeus length | Brunei | 43 | 2.58 | -0.169 | 0.313 | 0.591 |
| Clypeus length | USA | 21 | 2.45 | 0.571 | 1.789 | 0.752 |
| Inter ocular width | Malaysia | 65 | 3.22 | -0.189 | 0.210 | 0.371 |
| Inter ocular width | Argentina | 156 | 3.06 | 0.115 | 0.094 | 0.224 |
| Inter ocular width | Peru | 64 | 2.73 | -0.160 | 0.193 | 0.411 |
| Inter ocular width | Australia | 64 | 3.00 | -0.245 | 0.157 | 0.123 |
| Inter ocular width | Brunei | 42 | 2.56 | 0.119 | 0.147 | 0.421 |
| Inter ocular width | USA | 18 | 2.47 | 0.360 | 0.799 | 0.658 |
| **Weber’s length** | **Malaysia** | **80** | **3.20** | **-9.83E+13** | **3.390E+13** | **0.004** |
| Weber’s length | Argentina | 157 | 3.00 | 3.36E+13 | 1.438E+14 | 0.815 |
| Weber’s length | Peru | 70 | 0.06 | -3.37E+14 | 2.086E+14 | 0.111 |
| Weber’s length | Australia | 66 | 3.12 | 1.02E+13 | 5.056E+13 | 0.840 |
| Weber’s length | Brunei | 49 | 2.56 | 5.39E+13 | 2.844E+13 | 0.064 |
| Weber’s length | USA | 21 | 2.26 | -1.70E+14 | 8.209E+14 | 0.838 |
| **Whole body length** | **Malaysia** | **77** | **3.38** | **-0.207** | **0.056** | **0.0004** |
| **Whole body length** | **Argentina** | **157** | **3.05** | **0.103** | **0.047** | **0.029** |
| Whole body length | Peru | 59 | 2.70 | -0.045 | 0.082 | 0.585 |
| **Whole body length** | **Australia** | **66** | **3.08** | **-0.095** | **0.037** | **0.012** |
| Whole body length | Brunei | 40 | 2.54 | -0.029 | 0.086 | 0.739 |
| Whole body length | USA | 21 | 2.45 | 0.218 | 0.303 | 0.480 |
| Eye width | Malaysia | 66 | 3.23 | -2.192 | 1.531 | 0.157 |
| Eye width | Argentina | 153 | 3.05 | 0.457 | 0.379 | 0.228 |
| Eye width | Peru | 64 | 2.74 | -1.026 | 0.748 | 0.175 |
| Eye width | Australia | 64 | 2.99 | -0.61 | 0.542 | 0.265 |
| Eye width | Brunei | 42 | 2.62 | -0.568 | 0.698 | 0.420 |
| Eye width | USA | 18 | 2.47 | -2.343 | 3.880 | 0.554 |
| Eye position | Malaysia | 67 | 3.21 | -0.778 | 0.580 | 0.184 |
| **Eye position** | **Argentina** | **153** | **3.04** | **1.492** | **0.386** | **0.0001** |
| Eye position | Peru | 54 | 2.69 | -0.058 | 0.344 | 0.866 |
| Eye position | Australia | 64 | 2.99 | -0.196 | 0.411 | 0.634 |
| Eye position | Brunei | 39 | 2.60 | -0.140 | 0.305 | 0.648 |
| Eye position | USA | 19 | 2.47 | 1.27 | 2.557 | 0.625 |
| Pronotum width | Malaysia | 80 | 3.39 | -0.236 | 0.314 | 0.453 |
| Pronotum width | Argentina | 157 | 3.05 | -0.152 | 0.267 | 0.569 |
| Pronotum width | Peru | 64 | 2.73 | -0.107 | 0.132 | 0.421 |
| **Pronotum width** | **Australia** | **66** | **3.08** | **-0.260** | **0.125** | **0.042** |
| Pronotum width | Brunei | 43 | 2.58 | 0.165 | 0.147 | 0.269 |
| Pronotum width | USA | 21 | 2.45 | -0.134 | 1.574 | 0.933 |
| Hind femur length | Malaysia | 65 | 3.44 | -0.029 | 0.186 | 0.877 |
| Hind femur length | Argentina | 154 | 3.06 | -0.087 | 0.153 | 0.570 |
| Hind femur length | Peru | 63 | 2.73 | -0.025 | 0.128 | 0.843 |
| Hind femur length | Australia | 49 | 3.05 | -0.021 | 0.116 | 0.855 |
| Hind femur length | Brunei | 43 | 2.61 | 0.033 | 0.116 | 0.772 |
| Hind femur length | USA | 18 | 2.48 | 0.594 | 0.825 | 0.482 |
